# Supplementary material for: Facilitators and barriers to use rapid antigen test for SARS-CoV-2 among community-dwelling older adults in Hong Kong: A population-based cross-sectional random telephone survey
Source: PLOS Glob Public Health. 2023 Aug 8;3(8):e0002196. doi: 10.1371/journal.pgph.0002196 (PMC10409272; doi:10.1371/journal.pgph.0002196)
Supplement: Attachment — Submitted filename: Responses to comments_June23.docx [file pgph.0002196.s001.docx]

**Response to Reviewer comments**

PGPH-D-23-00780
Facilitators and barriers to use Rapid Antigen Test among community-dwelling older adults in Hong Kong: A Population-Based Random Telephone Survey
PLOS Global Public Health


**Journal Requirements**:

1. In the ethics statement in the Methods, you have specified that verbal consent was obtained. Please provide additional details regarding how this consent was documented and witnessed, and state whether this was approved by the IRB.

A: We have provided details regarding the procedures to obtain verbal consent.

“This study was a random telephone survey and there was no face-to-face contact between the interviewers and the participants. Therefore, verbal instead of written informed consent was obtained. The interviewers signed a form pledging the participants had been fully informed about the study. The first part of the form was a checklist to confirm whether the following contents have been introduced. They were: 1) research purpose, 2) research process, 3) main contents of the survey, 4) time required for completing the survey, 5) the rights of the participant, 6) the interviewer guaranteed that the denial to participate would have no consequences, and 7) the interviewer had explained the confidentiality of the research data. The interviewer then documented whether the participants fully understand the above-mentioned contents and whether they verbally expressed willingness to participate in the study in the same form. Questions raised by the participants about the study were also recorded in the form. Finally, the interviewers signed their names with date at the bottom of the form. Such procedures were approved by the Survey and Behavioral Research Committee of the Chinese University of Hong Kong. Same procedures to obtain verbal informed consent have been used in studies without face-to-face contacts [1-3].”

References

[1] Wang Z, et al. Illness representations on pneumonia and pneumococcal vaccination uptake among community-living Chinese people with high-risk conditions aged ≥65 years --- a population-based study. Human Vaccines & Immunotherapeutics, 2021; 17(5): 1455-1462

[2] Wang Z, et al. An online intervention promoting HIV testing service utilization among Chinese men who have sex with men during the COVID-19 pandemic: a quasi-experimental study. AIDS and Behavior, 2023; online ahead of print.

[3] Wang Z, et al. Parental acceptability of HPV vaccination for boys and girls aged 9-13 years in China --- a population-based study. Vaccine. 2018; 36(19): 2657-2665

2. We noticed you have some minor occurrence of overlapping text with the following previous publication(s), which needs to be addressed:
- [https://doi.org/10.3390/vaccines10060966](https://doi.org/10.3390/vaccines10060966)
In your revision ensure you cite all your sources (including your own works), and quote or rephrase any duplicated text outside the methods section. Further consideration is dependent on these concerns being addressed.

A: We have revised the manuscript to reduce overlapping with our published paper.

3. Please amend your online detailed Financial Disclosure statement. This is published with the article. It must therefore be completed in full sentences and contain the exact wording you wish to be published.

a) State the initials, alongside each funding source, of each author to receive each grant. For example: "This work was supported by the National Institutes of Health (####### to AM; ###### to CJ) and the National Science Foundation (###### to AM)."
b) State what role the funders took in the study. If the funders had no role in your study, please state: “The funders had no role in study design, data collection and analysis, decision to publish, or preparation of the manuscript.”

A: We have revised our financial disclosure statement accordingly:

“This work was supported by Health and Medical Research Fund, Food and Health Bureau, Hong Kong Special Administrative Region (19181152 to ZW). The funders had no role in study design, data collection and analysis, decision to publish, or preparation of the manuscript”.

4. Please ensure that the funders and grant numbers match between the Financial Disclosure field and the Funding Information tab in your submission form. Note that the funders must be provided in the same order in both places as well.

A: We have ensured that the funders and grant numbers are consistent between the Financial Disclosure Statement in the manuscript and that in the submission system.

5. Please update your online Competing Interests statement. If you have no competing interests to declare, please state: “The authors have declared that no competing interests exist.”

A: We have updated the completing interest statement accordingly.

6. Please provide separate figure files in .tif or .eps format only and remove any figures embedded in your manuscript file. Please also ensure that all files are under our size limit of 10MB.

For more information about how to convert your figure files please see our guidelines: [https://journals.plos.org/globalpublichealth/s/figures](https://journals.plos.org/globalpublichealth/s/figures)

A: We have removed the figures from the revised manuscript and uploaded them as separated files to the online submission system.

7. Please review your reference list to ensure that it is complete and correct. If you have cited papers that have been retracted, please include the rationale for doing so in the manuscript text, or remove these references and replace them with relevant current references. Any changes to the reference list should be mentioned in the rebuttal letter that accompanies your revised manuscript. If you need to cite a retracted article, indicate the article’s retracted status in the References list and also include a citation and full reference for the retraction notice.

A: We have crosschecked the reference list and ensured that it was complete and correct.

**Additional Editor Comments** (if provided):
Please respond to the comments made by the reviewers. In particular, please note the following:
1. I agree with Reviewer #1 that the term "significant other" is not a very specific term. Please replace this with a more specific term.

A: We agreed with the editor and reviewers. We have replaced “significant others” with “Hong Kong government and your children and/or other family members”.

2. I also agree that the title should be changed to specify COVID-19 or SARS-CoV-2.

A: We agreed with the editor and changed the title to: “Facilitators and barriers to use rapid antigen test for SARS-CoV-2 among community-dwelling older adults in Hong Kong: a population-based cross-sectional random telephone survey”.

3. One of the recommendations in STROBE is to describe clearly the study design (cohort, cross-sectional, etc.) in the title and/or abstract. Please specify that the study employed a cross-sectional study design in the abstract and Methods section of the main text.

A: We have highlighted the study design (cross-sectional) in the title, abstract and methods section of the revised manuscript.

4. I also share the same concern as Reviewer #2 regarding potential for selection bias in the current era of cell phone and landline use. Please clarify in Methods whether the database used for sampling contains both cell phone and landline use. Please also provide any information that supports the completeness of this database in covering all residents >=65 years age in Hong Kong. Please address this issue in Limitations, if completeness can not be verified.

A: We have clarified that the database used for sampling only contained landline phone numbers. In 2023, the residential landline penetration rate was 73.26% in Hong Kong [1]. The landline ownership was higher among elderly than that of the younger generation [1]. However, we agreed with the editor and the reviewer that it was a limitation that the database could not cover all residents in Hong Kong. We have highlighted this point in the limitation section of the revised manuscript.

Reference

[1] Office of the Communications Authority. Key communications statistics. Available at: <https://www.ofca.gov.hk/en/media_focus/data_statistics/key_stat/>. Accessed on June 21, 2023.


**Reviewers' comments**:
Reviewer's Responses to Questions

**Comments to the Author**

1. Does this manuscript meet PLOS Global Public Health’s [publication criteria](https://journals.plos.org/globalpublichealth/s/criteria-for-publication)? Is the manuscript technically sound, and do the data support the conclusions? The manuscript must describe methodologically and ethically rigorous research with conclusions that are appropriately drawn based on the data presented.

Reviewer #1: Yes

Reviewer #2: Yes

2. Has the statistical analysis been performed appropriately and rigorously?

Reviewer #1: Yes

Reviewer #2: Yes

3. Have the authors made all data underlying the findings in their manuscript fully available (please refer to the Data Availability Statement at the start of the manuscript PDF file)?

The [PLOS Data policy](https://journals.plos.org/globalpublichealth/s/data-availability) requires authors to make all data underlying the findings described in their manuscript fully available without restriction, with rare exception. The data should be provided as part of the manuscript or its supporting information, or deposited to a public repository. For example, in addition to summary statistics, the data points behind means, medians and variance measures should be available. If there are restrictions on publicly sharing data—e.g. participant privacy or use of data from a third party—those must be specified.

Reviewer #1: No

Reviewer #2: Yes

4. Is the manuscript presented in an intelligible fashion and written in standard English?

PLOS Global Public Health does not copyedit accepted manuscripts, so the language in submitted articles must be clear, correct, and unambiguous. Any typographical or grammatical errors should be corrected at revision, so please note any specific errors here.

Reviewer #1: Yes

Reviewer #2: Yes

5. **Review Comments to the Author**

Please use the space provided to explain your answers to the questions above. You may also include additional comments for the author, including concerns about dual publication, research ethics, or publication ethics. (Please upload your review as an attachment if it exceeds 20,000 characters)

**Reviewer #1**: Thank you for the opportunity to review this manuscript. The manuscript is well written. Below a few minor comments

A: Thank you for your comments. We have carefully addressed these comments.

1. Title: Please add that this is testing for SARS-CoV-2

A: We agreed with the reviewer and changed our title to “Facilitators and barriers to use rapid antigen test for SARS-CoV-2 among community-dwelling older adults in Hong Kong: a population-based cross-sectional random telephone survey”

2. Results:
Factors
….perceived significant others would support them using RAT (AOR: 1.66, 95%CI: 1.13, 2.43) had more behavioral intention…: can you rephrase this sentence. The term 'perceived significant others' is not clear - you mean individuals who had a life partner?

A: Sorry for the confusion. We have defined what “significant others” mean, and changed the paragraph to “... and perceived significant others (i.e., Hong Kong government and their children and/or other family members) would support them…”

3. Discussion:
'Hard to choose a reliable RAT kit is the main difficulty encountered by local older adults when using RAT' - you mean difficulty to choose? Please clarify the statement.

A: Sorry for the confusion. We have clarified the statement to “Difficulty in selecting a reliable RAT kit from the many brands available on the market was a main difficulty encountered by local older adults.

4. It is possible that the availability of free test kits increased someone's intention to test. Did the authors include this into their analyses? Is there any difference between before/after June 2022, when the free test kit program stopped?

A: Thank you for your suggestion. We performed additional analyses comparing level of intention before and after June 30, 2022, when the government stopped providing free RAT kits. The results showed that behavioral intention to use RAT kits with some symptoms was significantly lower after June 2022, as compared to the time before June 2022 (60% versus 88.2%, p<.001). However, the level of behavioral intention to use RAT kits without any symptoms was not statistically different before and after June 2022 (27.9% versus 13.3%, p=.22). We have elaborated the comparisons in the revised results and discussion sections.

5. Can you clarify what were the guideline from Hong Kong Dept of Health on testing with and without symptoms? Was it mandatory to test without symptoms? - Why would anyone want to test without any symptoms?

A: Sorry for the confusion. We have clarified the guideline on the use of RAT during the study period in the revised introduction session.

“In response to the fifth wave of the COVID-19 outbreak and the limited service capacity of NAATs, the Hong Kong Department of Health (HKDH) recommends the use of RAT [1]. Use of RAT is voluntary for people with and without COVID-19 symptoms. The guideline stated the procedures when people obtained positive results. First, all people with positive RAT results should report to the Centre for Health Protection within 24 hours via an online platform. Second, they are arranged for a door-to-door delivery of a nasal swab self-sampling kit by courier service for NAATs. Positive cases confirmed by NAATs are then triaged based on risk assessment and appropriate protocol taken and care given [1]. Since older adults are at higher risk of COVID-19, the government also recommend older adults to undergo frequent SARS-CoV-2 testing even if they did not have any symptoms for early detection and timely treatment [2]. Older adults who have received at least two doses of COVID-19 vaccination are recommended to undergo an RAT at least once per week, and those who were not fully vaccinated should do it at least three times a week [2].”

We have clarified that it was voluntary to test for SARS-CoV-2 regardless of symptoms. During the study period, the Hong Kong government recommended older adults to undergo frequent SARS-CoV-2 testing even they did not have any symptoms. The government stated that older adults had higher risk of COVID-19. Making good use of RAT kits to conduct regular testing allows early identification of infection, so that appropriate treatment could be provided in a timely manner. The government suggested that older adults who have received at least two doses of COVID-19 vaccination to undergo an RAT at least once per week, and those who were not fully vaccinated should do it at least three times a week [2]. It is expected that some older adults would follow the recommendation to use RAT without any symptoms.

References

[1] Hong Kong Department of Health. Rapid Antigen Test (RAT) for COVID-19. April 2022. [(accessed on 13 September 2022)]; Available online: <https://www.coronavirus.gov.hk/pdf/RapAgTest_FAQ_ENG.pdf>

[2] The Government of the Hong Kong Special Administrative Region. Government repeats appeal for elderly persons to undergo frequent COVID-19 testing. Available at: <https://www.info.gov.hk/gia/general/202205/04/P2022050400250.htm?fontSize=1>. Accessed on June 23, 2023

**Reviewer #2:** The study examined the behavioral intentions of older adults in Hong Kong to use rapid antigen tests (RAT) to monitor and test for COVID-19. The authors provide useful information about the acceptability of RAT among a high-risk population group, and offer recommendations to effectively support older adults, particularly during global health emergencies such as the COVID-19 pandemic. Overall, the paper is a good contribution to the literature and the development of public health programs. Below are a few recommendations for improvement.

A: Thank you for your comments. We have carefully addressed them in the revised manuscript.

1. Sample size planning: 18.2% of Hong Kong residents are aged 65 years and older. What is the total population?

A: We have provided the total population of residents aged 65 years or above in the revised manuscript (1.36 million in 2021).


2. Statistical analysis: In this statement "We then fitted a single logistic regression model involving all significant background characteristics and one independent variable of interest", please clarify "one independent variable of interest". Is this referring to the factors identified from the principal component analysis/exploratory factor analysis?

A: Sorry for the confusion. In this study, independent variables of interest referred to perceptions related to RAT based on the TPB, satisfaction of RAT promotional materials, and thoughtful consideration of the veracity of the information. In the adjusted analysis, the associations between one of the aforementioned independent variables and the dependent variables were investigated, after adjusting all significant background characteristics.


3. Results
i) In the first sentence on page 8, elaborate on what "compulsory testing" means. Does this refer to testing enforced by the government/public health officials?

A: Sorry for the confusion. Compulsory testing referred to testing enforced by the government. We have clarified this point in the revised manuscript.


4. ii) Among the 21.6% of RAT users who tested positive, it would also be interesting to see the percent breakdown of individuals who intended to use RAT regularly for health monitoring (without symptoms) vs those who intended to use RAT when symptomatic.

A: Thank you for your suggestion. Among individuals who intended to use RAT regularly for health monitoring, 20.8% had received positive RAT results in the past. Among those who intended to use RAT when symptomatic, 20.8% had received positive RAT results in the past.


5. Discussion
Given that TPB was the guiding framework of the study, it would be good to include a statement or two about the theory and tie that to the individual constructs (i.e., constructs that were found to be significantly associated with the outcome variables) prior to providing recommendations about how these results might inform future health interventions. The authors do partly highlight this in the "Implications" section, however, it would be good to connect to the TPB and also provide a few citations from the literature.

A: We agreed with the reviewer and included a statement about TPB at the beginning of the “implication session”:

“The TPB is a useful framework to understand factors associated with behavioral intention to use RAT, as three of its four constructs (i.e., positive attitudes, negative attitudes, and perceived subjective norm) were significantly associated with the dependent variables.”

6. Limitations: These are a couple of points to consider- adding to the issue of selection bias, might people's preference for cell phones over landlines be a potential study limitation, or are landlines still the preferred mode of communication among older adults in Hong Kong? Also, is it possible that some residents may be registered in the national "Do not call" list to avoid unsolicited calls, e.g., telemarketing?

A: In 2023, the residential landline penetration rate was 73.26% in Hong Kong [1]. The landline ownership was higher among elderly than that of the younger generation [1]. However, we agreed with the editor and the reviewer that it was a limitation that the database could not cover all residents in Hong Kong. We have highlighted this point in the limitation section of the revised manuscript.

Reference

[1] Office of the Communications Authority. Key communications statistics. Available at: <https://www.ofca.gov.hk/en/media_focus/data_statistics/key_stat/>. Accessed on June 21, 2023.

7. Additional comments: The writing can be improved for clarity. Below are a few suggestions that can be tweaked.
i) On page 5, the sentence "Interviewers screened the eligibility of prospective participants, briefed them about the study, and made guarantees of anonymity, their right to quit at anytime and refusal would have no consequences" can be written as "Participants were screened for eligibility, briefed about the study, and guaranteed anonymity. Participants were also assured that participation was voluntary and that opting-out of the study or declining to participate would have no consequences."

A: We have changed our statements based on reviewer’s suggestions.

8. "All informants had the experience of using RAT before." can be written as "All informants had prior experience using RAT."

A: Thank you for your suggestion. We made changes accordingly.

9. In the second paragraph on page 9, the first sentence, "Hard to choose a reliable RAT kit is the main difficulty encountered by local older adults when using RAT", can be rewritten as "Difficulty selecting a reliable RAT kit is the main challenge faced by local older adults".

Also, the last sentence in this paragraph can be rephrased from "Nonetheless, more targeted efforts in community education are still needed to eliminate the existing small proportion of older adults facing such difficulties of the uncertainty of what to do in case of positive RAT results" to "Nonetheless, additional targeted efforts in community education are needed to cater to older adults who may be unsure about the next course of action after receiving a positive RAT result."

A: We have changed our statements based on reviewer’s suggestions.

10. In the first paragraph on page 10, "never-users" can be replaced by "first-time users of RAT" or "individuals without prior experience using RAT"

A: Thank you for your suggestion. We made changes accordingly.

11. Please double check that in-text citations are in the right format and proper numbering, e.g., "Wang et al., 2022b" on page 6 is inconsistent with others.

A: Sorry for the error. We have carefully cross-checked the in-text citations in the revised manuscript.
